# ^1^H NMR metabolomic responses correlated to meat quality of Nile tilapia (*Oreochromis niloticus*) reared under combined dietary salt and water salinity conditions

**DOI:** 10.1016/j.fochx.2025.103235

**Published:** 2025-11-03

**Authors:** Samart Sai-Ut, Sarayut Watchasit, Sylvia Indriani, Nattanan Srisakultiew, Surintorn Boonanuntanasarn, Chatsirin Nakharuthai, Passakorn Kingwascharapong, Jaksuma Pongsetkul

**Affiliations:** a Department of Food Science, Faculty of Science, Burapha University, Chonburi 20131, Thailand; b Department of Chemistry, Faculty of Science, Burapha University, Chonburi 20131, Thailand; c School of Animal Technology and Innovation, Institute of Agricultural Technology, Suranaree University of Technology, Nakhon Ratchasima 30000, Thailand; d Department of Fishery Products, Faculty of Fisheries, Kasetsart University, Bangkok 10900, Thailand

**Keywords:** Nile tilapia, Salinity stress, Dietary salt, Meat quality, ^1^H NMR metabolomics

## Abstract

Freshwater scarcity has driven interest in culturing euryhaline species such as Nile tilapia (*Oreochromis niloticus*) in saline systems for sustainable aquaculture. This study examined the combined effects of dietary NaCl (2.5 % and 5.0 %) and water salinity (0, 15, 25 ppt) on tilapia muscle metabolome and meat quality using ^1^H NMR metabolomics. Fish fed 5.0 % NaCl at higher salinities showed reduced muscle protein, increased salt, elevated TBARS, and lower TVB-N (*P* < 0.05). Under these conditions, water-holding capacity improved, while hardness and chewiness decreased, suggesting softer texture. Sensory evaluation showed no differences in consumer acceptance (*P* > 0.05). Metabolomics revealed ten key metabolites (e.g., carnosine, tyrosine, betaine, glycogen, lactate) and seven enriched pathways linked to osmoregulation and energy metabolism. Multivariate analyses indicated dietary salt exerted greater influence than water salinity, highlighting the need for feed adaptation. Overall, results demonstrate physiological trade-offs of salinity adaptation and identify potential biomarkers for meat quality in saline aquaculture.

## Highlights

• ^1^H NMR revealed salinity-induced metabolic shifts in Nile tilapia.
• Dietary salt influenced meat metabolome and quality more than water salinity.
• High salinity (5 % NaCl, 25 ppt) elevated osmolytes and energy-related metabolites.
• Ten key metabolites and seven enriched pathways were identified.
• Muscle composition and traits (TVB-N, TBARS, texture) were significantly altered.

## Graphical abstract

**Figure:**
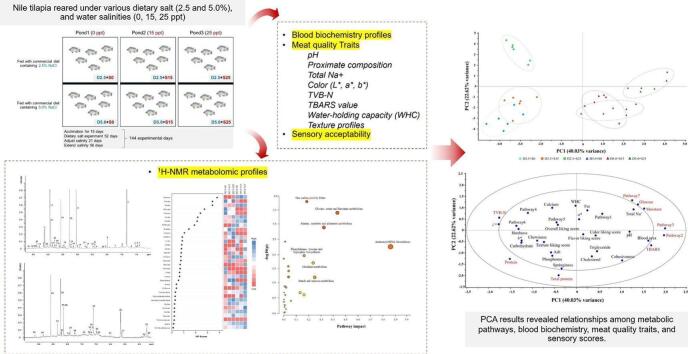

## 1 Introduction

The global aquaculture sector faces increasing pressure to sustainably meet the rising demand for high-quality animal protein amid rapid population growth and declining freshwater resources caused by climate change and competing industrial uses. These challenges have heightened interest in using brackish and saline water for aquaculture, with euryhaline species offering a promising solution due to their salinity tolerance (Musie & Gonfa, 2023). Nile tilapia (*Oreochromis niloticus*), one of the most widely cultured and economically significant finfish worldwide, is well suited to such systems, given its rapid growth, efficient feed utilization, and broad salinity tolerance (FAO, 2022). While typically farmed in freshwater, tilapia can tolerate salinities up to 10 ppt and beyond under proper management, making it a viable candidate for saline and brackish water aquaculture, especially in freshwater-limited regions (Dawood et al., 2022).

Salinity exposure, together with dietary salt supplementation, affects key physiological processes in fish, including osmoregulation, immune function, energy metabolism, and oxidative stress responses (Dildar et al., 2025; Hu et al., 2024; Huang et al., 2023). In Nile tilapia, elevated salinity impaired growth and feed efficiency, reduced lipid content, and induced oxidative stress, marked by increased antioxidant activity and lipid peroxidation (Gan et al., 2016). Dawood et al. (2022) further reported that tilapia maintained normal growth up to 10 ppt, but at 10–15 ppt growth declined, tissue damage occurred, stress markers rose, and antioxidant defenses weakened. At higher levels (20–30 ppt), salinity disrupted gut microbiota, increased intestinal permeability, impaired immune balance, and suppressed growth (Zhou et al., 2020). Dietary salt supplementation has shown promise in mitigating these effects. Nakajima and Sugiura (2016) demonstrated that up to 3.5 % dietary salt improved growth by enhancing osmoregulation and reducing osmotic stress, while Monier et al. (2025) found that 10 g NaCl/kg feed alleviated stress and enabled tolerance up to 24 g/L salinity. Beyond physiology, salinity adaptation also affects meat quality. Zhou et al. (2024) showed that culturing blue tilapia (*O. aureus*) in low brackish water (∼5 ppt) improved growth, muscle texture, nutrient profiles, and PUFA synthesis, thereby enhancing flesh quality as a cost-effective strategy. Likewise, Chen et al. (2024) reported that short-term salinity exposure enhanced biochemical traits, antioxidant activity, WHC, texture, nutritional value, and umami compounds in freshwater drum (*Aplodinotus grunniens*), supporting its use to improve meat quality. However, integrated studies linking these factors to metabolic shifts and comprehensive meat quality traits remain limited, especially using advanced metabolomic approaches. Despite these advances, few studies have simultaneously examined how salinity and dietary salt alter metabolomic profiles and their direct consequences for tilapia meat quality, leaving a critical gap for aquaculture optimization.

Proton nuclear magnetic resonance (^1^H NMR) spectroscopy-based metabolomics, combined with multivariate data analysis, is a powerful tool for profiling low-molecular-weight metabolites in biological tissues (Zhang et al., 2021). Its applications in aquaculture and meat science are well established: Hörterer et al. (2023) identified early metabolic changes in turbot fed alternative diets, Bodin et al. (2022) differentiated tuna species and storage conditions by metabolic profiles, Shen et al. (2021) linked taurine supplementation to improved nutrient utilization and growth in tilapia, and Indriani et al. (2024) revealed metabolomic signatures underlying flavor differences in chicken breeds. These examples underscore the value of ^1^H NMR metabolomics in identifying quality biomarkers and elucidating metabolic pathways linked to meat quality and consumer-relevant traits beyond conventional physiological measures. Building on this foundation, the present study investigates the interactive effects of dietary salt supplementation and water salinity on the metabolomic profiles and meat quality of Nile tilapia. Using ^1^H NMR spectroscopy-based metabolomic profiling, the study characterizes muscle tissue alterations across varying salinity conditions, alongside assessments of physicochemical quality and sensory attributes. Additionally, principal component analysis (PCA) was applied to explore correlations among key metabolite pathways, quality indicators, and sensory profiles to identify potential biomarkers associated with salinity-induced physiological adaptations and consumer acceptability. This integrated approach aims to offer mechanistic explanations into the effects of salinity on tilapia metabolism and quality, supporting optimized aquaculture practices and sustainable product outcomes.

## 2 Materials and methods

### 2.1 Experimental fish and fish culture

The research was carried out in accordance with animal experimentation regulations and the Guidelines for the Use of Animals in Research, as recommended by Thailand's National Research Council. All procedures described herein were supervised and approved by Suranaree University of Technology's Animal Ethics Committee (approval no. SUT-IACUC-4930030/2023).

**Fig. 1:**
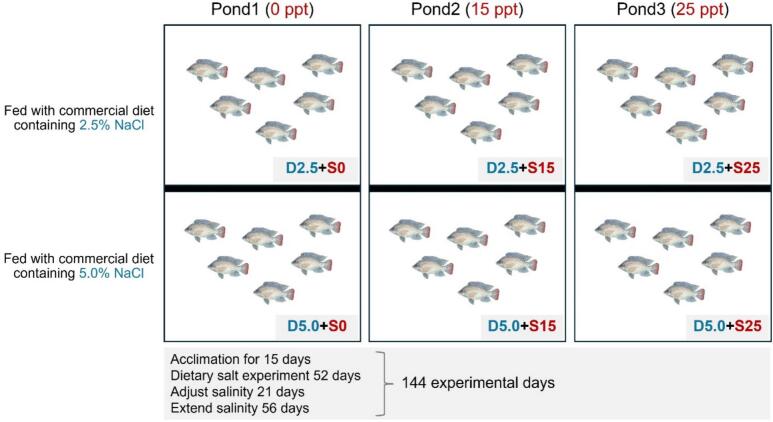
The experiment design: A schematic of the pond layout.

To mimic the coastal aquaculture system, water salinities of 15 ppt (S15) and 25 ppt (S25) were selected for comparison with the freshwater culture system at 0 ppt (S0). A previous report revealed that a 5 % salt-enriched diet significantly improved nutrient digestibility (Hallali et al., 2018). The commercial diet (basal diet) used in this study contained 2.5 % NaCl. Therefore, this study employed two different levels of dietary salt: 2.5 % (D2.5) and 5 % (D5.0). Diet D5.0 was prepared by homogeneously spraying a NaCl solution onto the basal diet, followed by drying at room temperature (25 °C) for 12 h. A schematic of the experimental design and pond layout, showing six experimental groups: D2.5 + S0 (control), D5.0 + S0, D2.5 + S15, D5.0 + S15, D2.5 + S25, and D5.0 + S25, is presented in Fig. 1. Three experimental ponds (5 m × 10 m × 1 m; water level 0.8 m) were used for three different salinity levels: 0 ppt (S0), 15 ppt (S15), and 25 ppt (S25). Inside each pond, nets were used to divide the pond into two halves, with fish in each section fed diets supplemented with two different levels of salt: 2.5 % (D2.5) and 5 % (D5.0). To minimize the effect of pond-to-pond (environmental) variability, a completely randomized design for communal testing with six replications was used within the same partition (treatment). Nile tilapia (*O. niloticus*), weighing 50–80 g, were collected from farms in Nakhon Pathom Province, Thailand, and transferred to Suranaree University of Technology Farm (SUT Farm). This study maintained the optimum stocking density (2.32 kg/m^3^) for fish reared in an intensive aquaculture system with aeration (Komal et al., 2024), in conjunction with the SUT Farm practical method (10 fish/m^2^ in ponds with continuous aeration). All fish were tagged with PIT tags, and 30 fish per replication were randomly distributed into each communal pond, resulting in a total of 180 fish per communal partition, with an initial stocking density of 0.5 kg/m^3^. All fish were acclimated for 15 days and fed a commercial diet (32 % crude protein, 6 % crude fat 2.5 % NaCl) under natural light (∼12 h light: ∼12 h dark) prior to the experiment at SUT Farm.

After acclimation, fish in the D2.5 group continued to be fed with the commercial diet (2.5 % NaCl), while those in the D5.0 group were fed a diet containing 5.0 % NaCl (D5.0), at a feeding rate of 2 % of body weight, twice daily. The proximate composition of the diets is presented in **Table S1.** After 52 days of feeding, fish were assigned to three water salinity levels: 0, 15, and 25 ppt. During this phase, water salinity gradually increased by 2 ppt every two days until the target levels were reached over a period of 21 days. All six experimental groups (D2.5 + S0, D2.5 + S15, D2.5 + S25, D5.0 + S0, D5.0 + S15, D5.0 + S25) were then reared for an additional 56 days until reaching marketable size (404.29–492.21 g). Throughout the 144-day experimental period, water quality in the culture system was maintained within optimal limits by exchanging one-third of the water and providing aeration every three days. Water quality parameters were measured six times per week, at 8:00 a.m. and 5:00 p.m. on Mondays, Wednesdays, and Fridays. All recorded values remained within acceptable ranges throughout the study period, including air temperature (26.80 ± 1.35 °C), water temperature (28.05 ± 1.01 °C), dissolved oxygen (6.06 ± 0.47 mg/L), and pH (7.75 ± 0.32).

### 2.2 Sample collection and preparation

Throughout the 144-day experimental period, fish were sampled 5 h after feeding, as this time corresponds with peak postprandial glycemia (Boonanuntanasarn et al., 2018). Two fish from each replicate (*n* = 12 fish per experimental treatment) were randomly selected and euthanized using clove oil at a concentration of 1.5 g/mL. Blood samples were collected from the caudal vein using a hypodermic syringe and mixed with K₂EDTA (1.5 mg/mL of blood) as an anticoagulant, then kept on ice. Plasma was obtained by centrifugation of the K₂EDTA-treated blood at 5000 ×*g* for 10 min at 4 °C and stored at −80 °C for subsequent blood chemistry analysis. After blood collection, muscle tissue samples were harvested, immediately frozen, and stored at −40 °C for meat quality analysis within one month. For metabolome analysis, 50 g of each sample were powdered using liquid nitrogen, homogenized with a blender (National, Tokyo, Japan), and stored at −80 °C until analysis.

### 2.3 Determination of metabolomic profiling using ^1^H NMR

#### 2.3.1 Metabolite extraction and ^1^H NMR measurement

Proton nuclear magnetic resonance (^1^H NMR) was used to perform metabolomic profiling of the samples, following modified protocols based on Li et al. (2014) for metabolite extraction and Sai-Ut et al. (2024) for NMR acquisition. For sample extraction, 100 mg of fish powder was dissolved in 2000 μL of 80 % (*v/v*) deuterium oxide containing 80 mM phosphate buffer (pH 7.4) and 0.01 mM 3-(trimethylsilyl)propionic-2,2,3,3-d₄ acid sodium salt (TSP) as the internal standard. The mixture was sonicated for 1 min and centrifuged at 10,000 rpm for 5 min. The supernatant was filtered through a 0.45 μm syringe filter, and 600 μL of the filtrate was transferred into a 5-mm NMR tube. All ^1^H-qNMR experiments were performed on a Bruker Avance III HD 400 MHz NMR spectrometer equipped with a 5 mm CryoProbe Prodigy (double resonance broadband observed with ^19^F probe) at 25 °C. The ^1^H-qNMR spectra of the fish powder water extract were acquired using the following parameters: pulse program *zg*; relaxation delay, 60 s; pulse width, 12.25 μs; number of scans, 32; data points, 65 K; sweep width, 24 ppm; and spectral center, 4.7 ppm. Line broadening (LB = 0.3 Hz) was applied during processing.

#### 2.3.2 Metabolite identification, quantification, and pathway analysis

The Chenomx Processor software (Chenomx Inc., Edmonton, Canada) was used for peak alignment and baseline normalization. All the spectra were referenced to the internal standard TSP and matched against the Chenomx Compound Library. The databases of the Human Metabolome Database (HMDB), Madison-Qingdao Metabolomics Consortium Database (MMCD), Kyoto EnFoods cyclopedia of Genes and Genomes (KEGG), as well as Biological Magnetic Resonance Data Bank (BMRB) were used. Metabolite concentrations were normalized to sample weight before multivariate analysis.

Statistical analysis of metabolite spectra was performed using the MetaboAnalyst 6.0 platform (http://www.metaboanalyst.ca/). Partial least squares discriminant analysis (PLS-DA) was applied to reduce data dimensionality, and model performance was evaluated by leave-one-out cross-validation. Model fit and predictive ability were assessed using the coefficient of determination (*R*^2^) and predictive ability (*Q*^2^), respectively. Variable importance in projection (VIP) scores was calculated to identify metabolites contributing to group separation, with VIP > 1 indicating significance. For each significant metabolite, one-way ANOVA followed by Tukey's post hoc test was conducted at *P* < 0.05 using SPSS (v25, IBM, Armonk, NY, USA). For pathway analysis, the *Danio rerio* (zebrafish) KEGG library within MetaboAnalyst 6.0 was used, and pathways with *P* < 0.05 were considered significant, consistent with the exploratory nature of the study.

### 2.4 Determination of blood biochemistry profiles

Blood metabolites, including glucose, triglycerides, cholesterol, total protein, blood urea nitrogen (BUN), calcium, and phosphorus, were analyzed following Luo et al. (2025). Briefly, glucose was quantified using the Trinder reaction and triglycerides by the glycerol-3-phosphate oxidase–sodium N-ethyl-N-(3-sulfopropyl) m-anisidine method. Cholesterol was measured by the enzymatic colorimetric cholesterol oxidase–phenol + aminophenazone (CHOD-PAP) technique. Total protein was determined by the Biuret method, and BUN by a modified indophenol colorimetric method. Calcium and phosphorus were measured using an arsenazo III colorimetric method and a molybdovanadate colorimetric method, respectively.

### 2.5 Determination of meat quality traits

The pH was measured using a pH meter (Sartorius, Göttingen, Germany) after homogenizing 1 g of fish meat with 100 mL of distilled water. Proximate composition, including moisture, protein, fat, and ash content, was determined using the AOAC method (AOAC, 2000). Total Na^+^ was determined using the in-house method TE-CH-134 Briefly, fish samples (∼1 g) were digested with concentrated nitric acid and hydrogen peroxide using a microwave-assisted digestion system. The resulting clear solution was diluted to a known volume with deionized water and analyzed for sodium using inductively coupled plasma optical emission spectrometry (ICP-OES) at Central Laboratory (Thailand) Co., Ltd. (CLT), which is certified to ISO/IEC 17025:2017. Color was measured by taking at least five readings at different locations on each sample using an automatic colorimeter (ColourFlex, HunterLab, Reston, VA, USA), and the results were expressed as L* (lightness), a* (redness/greenness), and b* (yellowness/blueness). Total volatile base nitrogen (TVB-N) content was estimated by the Conway micro-diffusion method and expressed as mg N/100 g, while thiobarbituric acid reactive substance (TBARS) values were calculated with malonaldehyde bis(dimethylacetal) (0–2 ppm) used as standards and expressed as mg/kg using the method proposed by Pongsetkul et al. (2022). WHC was expressed as the percentage of water retained after centrifugation at 1000 ×*g* for 5 min at 4 °C. For texture analysis, fish meat was cooked sous-vide in a water bath at 85 °C for 10 min and then measured using a texture analyzer (TA.XTplus; Stable Micro Systems, Godalming, UK) equipped with a cylindrical probe (P-50, 50 mm diameter). Test parameters were TPA macro mode, pre-test speed 1 mm/s, test speed 1.0 mm/s, post-test speed 5.0 mm/s, 50 % strain, 5 s interval, and 5 g trigger force. Hardness (N), chewiness (N^⁎^mm), springiness (mm), and cohesiveness (ratio) were calculated following the method of Kingwascharapong et al. (2024) with slight modifications.

### 2.6 Sensory evaluation

Fifty untrained panelists (aged 19–35 years; 20 men, 30 women) were asked to evaluate color, texture, flavor, and overall liking using a nine-point hedonic scale (1 = dislike extremely, 9 = like extremely). The study protocol was approved by the Ethics Committee on Human Research, Suranaree University of Technology (SUT), Thailand (Document ID: EC-67-189). All procedures were designed to protect the rights and privacy of participants, and informed consent was obtained from all participants. For sample preparation, fish was cooked sous-vide in a water bath at 85 °C for 10 min, cut into 1 × 2 × 1 cm^3^ cubes, and served with Thai seafood dipping sauce on white paper plates. Sensory evaluation was conducted in individual booths under fluorescent white light. Panelists were instructed to rest for 2 min after every three samples to minimize sensory fatigue.

### 2.7 Statistical analysis

Data are expressed as mean ± standard deviation (SD). Statistical differences among samples were evaluated using one-way analysis of variance (ANOVA) followed by Tukey's post hoc test, with significance defined at *P* < 0.05. Analyses were performed using SPSS software (v25, IBM, Armonk, NY, USA). Principal component analysis (PCA) was conducted to explore the relationship between differential metabolic pathways, blood biochemistry profile, meat quality traits, and sensory scores of samples, using Unscrambler® X software (v10.1, Camo Analytics, Oslo, Norway).

## 3 Results and discussion

### 3.1 Blood biochemistry profiles

Blood biochemical parameters serve as critical indicators of physiological and metabolic responses in fish exposed to environmental and nutritional stressors. In this study, plasma metabolites of Nile tilapia were analyzed to assess osmoregulatory and metabolic changes under different salinity levels (0, 15, and 25 ppt) and dietary salt supplementation (2.5 % and 5.0 %). No significant differences (*P* > 0.05) in plasma triglycerides, cholesterol, total protein, calcium, or phosphorus concentrations were observed among treatments (Table 1), suggesting that lipid metabolism and mineral homeostasis remained largely unaffected by either salinity or dietary salt variations. These results indicate effective physiological regulation of mineral balance and lipid mobilization under osmotic stress, reflecting an adaptive trait characteristic of euryhaline species. However, plasma glucose concentrations significantly increased (*P* < 0.05) with both elevated salinity and higher dietary salt intake. The highest glucose levels (4.76 ± 0.33 mmol/L) were observed in D5.0 + S25, indicating a synergistic effect of dietary and environmental salt stress. The result was consistent with Liu et al. (2025), who reported that tilapia reared in saline-alkaline water exhibited significantly elevated plasma glucose levels compared to those in freshwater. Similarly, Dawood et al. (2022) reported that blood glucose levels in Nile tilapia increased markedly at salinities of 10 and 15 ppt. Elevated glucose levels reflect the activation of gluconeogenesis and glycogenolysis, metabolic pathways that are commonly upregulated during stress, likely mediated by cortisol (Dildar et al., 2025). Glucose mobilization supports the increased energy demands required for active osmoregulation in hyperosmotic environments (Pan et al., 2023). Blood urea levels were elevated in fish reared at 25 ppt with 5.0 % dietary NaCl supplementation, as the D5.0 + S25 group showed significantly higher levels (*P* < 0.05) than the other groups, which did not differ significantly from each other (*P* > 0.05). This elevation indicated enhanced protein catabolism and nitrogenous waste production, representing adaptive responses to salinity stress (Dildar et al., 2025). Urea, in conjunction with free amino acids, functions as an organic osmolyte that helps regulate cellular osmotic balance (Burg & Ferraris, 2008). Elevated glucose and urea concentrations under high salinity conditions reflect the metabolic cost of homeostatic maintenance, which, if sustained, could compromise growth performance and fillet quality. While water salinity imposes osmotic stress on osmoregulatory organs such as the gills and kidneys, through direct effects of external ionic concentrations that increase ion exchange and metabolic energy demands, our overall findings indicated that dietary salt exerted a greater influence than water salinity on metabolic profiles and fillet quality, consistent with PCA results and downstream analyses.

**Table 1:** Blood biochemistry profiles (mmol/L) of tilapia meat reared under combined dietary salt and water salinity conditions.

**Parameter**	**D2.5 + S0**	**D2.5 + S15**	**D2.5 + S25**	**D5.0 + S0**	**D5.0 + S15**	**D5.0 + S25**
Glucose	4.00 ± 0.14^b^	4.52 ± 0.45^ab^	4.34 ± 0.28^ab^	4.23 ± 0.29^ab^	4.29 ± 0.26^ab^	4.76 ± 0.33^a^
Triglyceride	2.87 ± 0.30	3.02 ± 0.30	2.86 ± 0.41	2.94 ± 0.06	3.27 ± 0.41	2.95 ± 0.11
Cholesterol	3.10 ± 0.03	3.09 ± 0.05	3.02 ± 0.11	3.15 ± 0.05	3.08 ± 0.06	3.07 ± 0.02
Total protein⁎	4.83 ± 0.52	4.81 ± 0.10	4.38 ± 0.52	4.81 ± 0.40	4.63 ± 0.29	4.55 ± 0.47
Blood urea	0.19 ± 0.02^b^	0.19 ± 0.01^b^	0.18 ± 0.02^b^	0.19 ± 0.02^b^	0.20 ± 0.01^b^	0.23 ± 0.01^a^
Calcium	7.53 ± 0.17	7.55 ± 0.47	7.89 ± 0.11	7.42 ± 0.39	7.67 ± 0.21	7.47 ± 0.14
Phosphorus	3.15 ± 0.33	3.16 ± 0.34	2.84 ± 0.43	3.05 ± 0.45	3.35 ± 0.72	2.77 ± 0.22

Mean ± SD.

Different lowercase letters in the same row indicate significant differences (*P* < 0.05).

⁎ Reported as g/dl.

### 3.2 Meat quality traits

According to Table 2, dietary salt significantly impacted the pH of tilapia fillets, with the 5.0 % NaCl groups exhibited notably higher pH compared to those fed 2.5 % NaCl (*P* < 0.05). Although all treatments stayed within the normal pH range for fresh tilapia (6.0–6.8), the observed elevation might reflect delayed post-mortem glycolysis. High dietary salt intake can induce pre-slaughter stress, depleting muscle glycogen reserves and reducing lactic acid accumulation following death, consequently resulting in elevated ultimate pH values. Proximate analysis revealed that fillets from the 5.0 % NaCl groups had significantly lower crude protein content (14.93–15.85 %) than those from the 2.5 % NaCl groups (16.09–17.41 %) (*P* < 0.05). At the same time, these higher salt-fed groups exhibited significantly higher moisture and fat levels (*P* < 0.05). The reduced protein content might reflect a protein-sparing mechanism, where osmotic stress shifts energy metabolism toward lipid and carbohydrate utilization, while the increased fat deposition might stem from altered metabolic pathways under salt stress (Su et al., 2023). Fish fed 5.0 % dietary salt had higher fat content (3.88–4.06 %) than those fed lower salt levels (3.24–3.81 %) (*P* < 0.05). This finding contrasted with Gan et al. (2016), who reported higher whole-body crude lipid content in Nile tilapia reared in freshwater and low salinity compared to fish exposed to 16 and 24 ppt salinity. Nevertheless, the observed compositional changes here aligned with broader evidence that elevated salinity or dietary salt altered energy allocation and body composition in tilapia and seabass (Gan et al., 2016; Liu et al., 2025). While reduced protein content may negatively affect the nutritional value of tilapia meat cultured under salted conditions, the higher fat accumulation could partly offset this, as fish fatty acids are generally beneficial to health and may also contribute to desirable traits such as flavor and odor when the fish is consumed. In contrast, ash and carbohydrate contents did not differ significantly among treatments (*P* > 0.05), suggesting stable mineral balance and non-structural carbohydrate levels under the experimental conditions. The total Na^+^ content in muscle tissues increased progressively with both higher water salinity and dietary salt intake, with the highest concentrations observed in the D5.0 + S25 group (*P* < 0.05). This reflected efficient ion uptake and accumulation under hyperosmotic conditions a well-characterized adaptation of euryhaline fish to maintain osmotic equilibrium (Edwards and Marshall, 2012). The Na^+^ levels in all samples remained within the acceptable safety limits for human consumption, as excessive dietary sodium (>100 mg/100 g fish muscle) may pose concerns for hypertensive individuals (WHO, 2012). Nonetheless, the elevated Na^+^ content compared to normal freshwater-cultured tilapia suggested that post-harvest processing or culinary strategies might be necessary to manage sodium levels in the final consumer products.

**Table 2:** Meat quality characteristics and sensory scores of tilapia meat reared under combined dietary salt and water salinity conditions.

**Parameter**	**D2.5 + S0**	**D2.5 + S15**	**D2.5 + S25**	**D5.0 + S0**	**D5.0 + S15**	**D5.0 + S25**
pH	6.58 ± 0.12^c^	6.72 ± 0.08^bc^	6.70 ± 0.08^bc^	6.95 ± 0.06^a^	6.88 ± 0.10^ab^	6.80 ± 0.09^ab^
Proximate compositions (%)						
Moisture	75.90 ± 0.93^ab^	75.15 ± 0.40^b^	76.69 ± 0.36^a^	77.78 ± 0.67^a^	76.16 ± 1.50^a^	78.56 ± 1.75^a^
Protein	17.41 ± 0.39^a^	16.92 ± 0.88^ab^	16.09 ± 0.42^b^	14.93 ± 1.24^c^	15.85 ± 0.99^bc^	15.02 ± 0.29^c^
Fat	3.24 ± 0.21^c^	3.51 ± 0.18^b^	3.81 ± 0.24^ab^	4.02 ± 0.24^a^	4.06 ± 0.15^a^	3.88 ± 0.13^a^
Ash	2.67 ± 0.47	3.04 ± 0.64	2.48 ± 0.67	2.28 ± 0.25	2.91 ± 0.48	2.01 ± 0.65
Carbohydrate	0.78 ± 0.18	0.98 ± 0.34	0.93 ± 0.22	0.69 ± 0.10	0.97 ± 0.20	0.63 ± 0.39
Total Na^+^ (mg/kg)	416.95 ± 20.34^c^	518.02 ± 55.84^bc^	588.25 ± 56.84^ab^	463.23 ± 47.57^c^	520.87 ± 81.93^bc^	685.25 ± 109.09^a^
Color						
L*	47.66 ± 1.28^ab^	49.22 ± 0.66^a^	49.58 ± 1.09^ab^	44.27 ± 1.25^c^	46.88 ± 2.02^bc^	43.25 ± 0.99^c^
a*	−3.67 ± 0.31	−3.99 ± 0.50	−2.94 ± 0.52	−3.53 ± 0.39	−3.68 ± 0.25	−3.01 ± 0.36
b*	6.62 ± 0.43	5.99 ± 0.45	6.23 ± 0.50	6.05 ± 0.44	6.03 ± 0.39	5.98 ± 0.32
TVB-N (mg/100 g)	7.92 ± 0.50^a^	8.39 ± 0.36^a^	8.77 ± 0.30^a^	6.20 ± 0.35^b^	5.01 ± 0.41^c^	5.05 ± 0.23^c^
TBARS (mg/kg)	0.22 ± 0.04^b^	0.23 ± 0.03^b^	0.19 ± 0.06^b^	0.20 ± 0.04^b^	0.26 ± 0.05^ab^	0.33 ± 0.06^a^
WHC (%)	84.66 ± 0.73^b^	84.04 ± 0.70^b^	85.26 ± 0.64^b^	85.01 ± 1.01^ab^	87.09 ± 0.98^a^	86.22 ± 0.35^a^
Texture profiles						
Hardness (N)	695.22 ± 50.16^abc^	701.68 ± 43.92^ab^	777.31 ± 44.87^a^	603.96 ± 52.02^c^	648.28 ± 53.53^bc^	600.90 ± 40.93^c^
Springiness (mm)	0.73 ± 0.02	0.75 ± 0.04	0.69 ± 0.05	0.69 ± 0.05	0.75 ± 0.05	0.72 ± 0.04
Chewiness (N^⁎^mm)	182.70 ± 8.05^a^	184.19 ± 10.02^a^	171.63 ± 9.09^a^	145.86 ± 15.45^b^	179.90 ± 10.11^a^	168.73 ± 9.06^ab^
Cohesiveness	0.36 ± 0.03	0.35 ± 0.03	0.32 ± 0.04	0.35 ± 0.05	0.37 ± 0.02	0.39 ± 0.04
Sensory liking score						
Color	7.23 ± 0.25	7.55 ± 0.33	7.07 ± 0.46	7.17 ± 0.44	7.58 ± 0.32	7.94 ± 0.50
Texture	7.63 ± 0.44	7.70 ± 0.40	7.29 ± 0.39	7.55 ± 0.51	7.38 ± 0.39	7.20 ± 0.34
Flavor	7.05 ± 0.35	7.23 ± 0.39	7.07 ± 0.42	7.51 ± 0.33	7.09 ± 0.38	7.07 ± 0.38
Overall	7.20 ± 0.46	7.21 ± 0.40	7.35 ± 0.29	7.39 ± 0.45	7.04 ± 0.51	7.08 ± 0.49

Mean ± SD.

Different lowercase letters in the same row indicate significant differences (*P* < 0.05).

Color analysis showed significant differences in L* values (lightness), while a* (redness) and b* (yellowness) values did not differ among samples (*P* > 0.05). Fish fed 5.0 % NaCl had lower L* values (43.25–46.88) compared to those fed 2.5 % NaCl (47.66–49.58) (*P* < 0.05). This reduction in lightness might be attributed to increased muscle moisture and altered WHC, which reduced light scattering by tightening muscle fiber packing and decreasing air spaces, thereby lowering reflectance (Erikson & Misimi, 2008). Higher muscle salt content might also affect myofibrillar protein conformation and pigment binding, thereby contributing to color changes. TVB-N, composed mainly of ammonia, dimethylamine, and trimethylamine, indicates meat spoilage from microbial and enzymatic breakdown of muscle nitrogen and reflects deterioration in odor, flavor, and safety (Indriani et al., 2024). The 5.0 % NaCl groups showed significantly lower TVB-N levels (5.01–6.20 mg/100 g) than the 2.5 % NaCl groups (7.92–8.77 mg/100 g) (*P* < 0.05), suggesting that higher dietary salt more effectively suppressed microbial activity or endogenous enzymes, thereby reducing protein breakdown. These findings align with Ringø et al. (2022) who stated that high salinity in feed modulates the gut microbiota by promoting salt-tolerant probiotics (e.g., *Bacillus* spp., *Lactobacillus* spp.) and suppressing pathogenic bacteria such as *Aeromonas* spp., thereby extending the shelf life of the meat. Notably, all TVB-N values remained well below the 15–20 mg/100 g freshness threshold, indicating good quality and freshness of the samples (FAO, 1995). TBARS, an indicator of lipid oxidation, was significantly elevated in the D5.0 + S25 group (*P* < 0.05), correlating with higher muscle fat content and its potential susceptibility to oxidation; however, values remained below the widely accepted freshness threshold of 2 mg MDA/kg (Indriani et al., 2024), indicating that fillet quality was still acceptable, though high dietary salt and salinity accelerated lipid oxidation, potentially affecting flavor and odor. The results suggested that such conditions may contribute to unique sensory characteristics during storage or cooking; despite suppressing TVB-N, they promoted TBARS accumulation. Regarding texture quality, WHC and texture profiles were evaluated. Notably, the D5.0 + S15 and D5.0 + S25 groups showed significantly higher WHC than others (*P* < 0.05), likely due to increased moisture content and ionic strength. Salt ions enhance protein solubility and promote myofibrillar swelling, strengthening the muscle's ability to retain water (Kang et al., 2021; Wu et al., 2016). Improved WHC not only increases product yield but also improves sensory juiciness, a desirable trait for both processors and consumers (Pongsetkul et al., 2024). Texture analysis of sous-vide cooked fillets showed significantly reduced hardness and chewiness in the 5.0 % NaCl groups (*P* < 0.05). These changes may result from protein denaturation and structural weakening due to osmotic stress during rearing, leading to a softer, more easily chewable texture when cooking or storing under the same conditions, compared to fish raised in freshwater. High salinity could increase metabolic demand for salt excretion, leading to stress that alters growth rates and muscle structure and ultimately affects texture. While reduced hardness may enhance tenderness, excessive softness could compromise texture acceptability depending on consumer preference. However, no significant differences were observed in springiness and cohesiveness among groups (*P* > 0.05). Overall, the results suggested that culturing tilapia in high salinity conditions can alter meat quality, particularly flavor and texture, which points to the potential uniqueness of fish reared or bred under such conditions.

### 3.3 Sensory evaluation

Despite measurable differences in meat composition, color, texture, and biochemical properties across salinity and dietary salt treatments, no significant differences were observed in sensory scores for color, texture, flavor, or overall liking among all groups (*P* > 0.05) (Table 2). This lack of sensory discrimination could be due to the buffering effect of sous-vide cooking, which stabilized flavor and texture, or because changes in physicochemical traits did not exceed perceptual thresholds. These findings were consistent with Carmo et al. (2025), who reported no significant differences in taste, texture, juiciness, odor, or color scores (*P* > 0.05) between tilapia raised in brackish and freshwater; however, overall sensory impression was significantly higher (*P* < 0.05) for brackish water-raised fillets. In this study, all fillet samples received scores above the acceptability threshold; on a 9-point hedonic scale, a mean score of 7.00 or higher generally indicates high sensory quality. These suggested that physiological and biochemical changes induced by elevated salinity and dietary salt did not translate into perceptible differences in sensory quality detectable by consumers, with products still rated as high quality. This was particularly noteworthy given that several physicochemical traits (e.g., pH, fat content, TBARS, and texture) were significantly altered under the harsher salt conditions. Importantly, the sensory results supported the feasibility of rearing Nile tilapia under high salinity, such as the D5.0 + S25 treatment, without compromising consumer-perceived quality. Nevertheless, although sensory quality remained acceptable under this harsh condition, the reduction in protein content and increase in fat oxidation observed in this group may negatively impact nutritional value, flavor stability, and shelf life; factors not assessed in the present study and recommended for future investigation. This finding highlights the prospect of expanding tilapia aquaculture into brackish or saline environments (up to 25 ppt) with elevated dietary salt as a sustainable production strategy, especially in regions with limited freshwater resources. Maintaining acceptable sensory quality under osmotic stress enhances product marketability while supporting economic and environmental goals in aquaculture development.

### 3.4 Metabolomic profiles

**Fig. 2:**
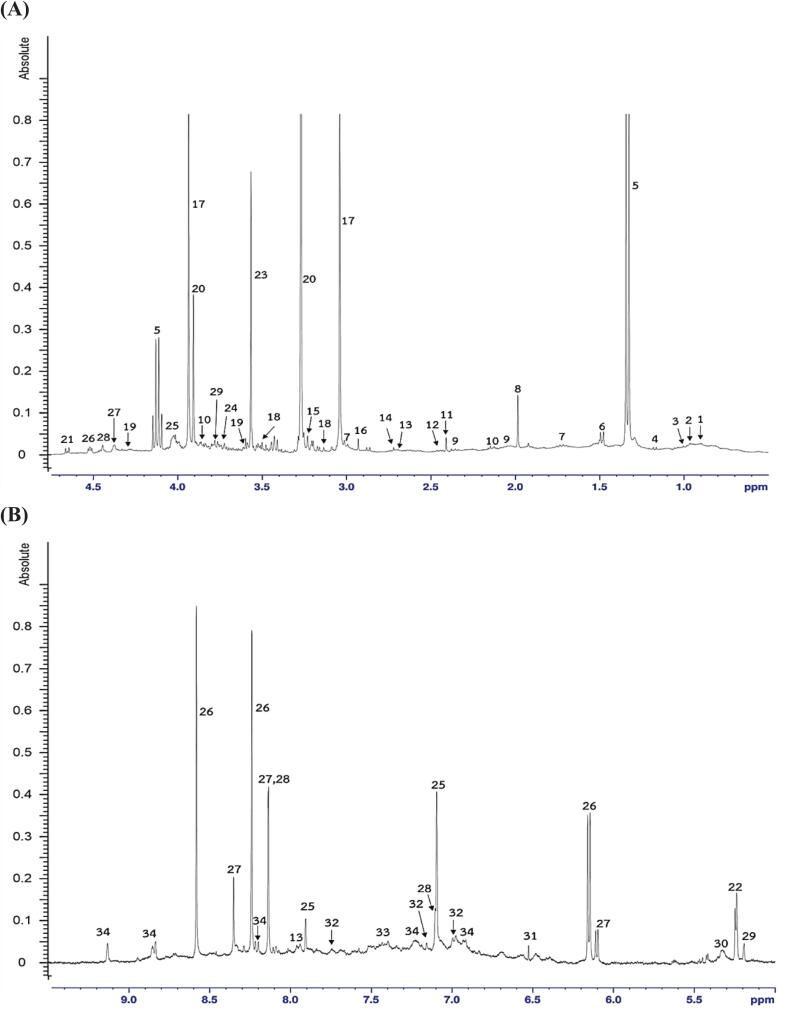
Representative 400 MHz water-suppression (noesygppr1d) ^1^H NMR spectrum (**A**: δ0.50–4.75 and **B**: δ5.0–10.00 ppm) of tilapia meat reared under combined dietary salt and water salinity conditions (34 metabolites).

**Fig. 3:**
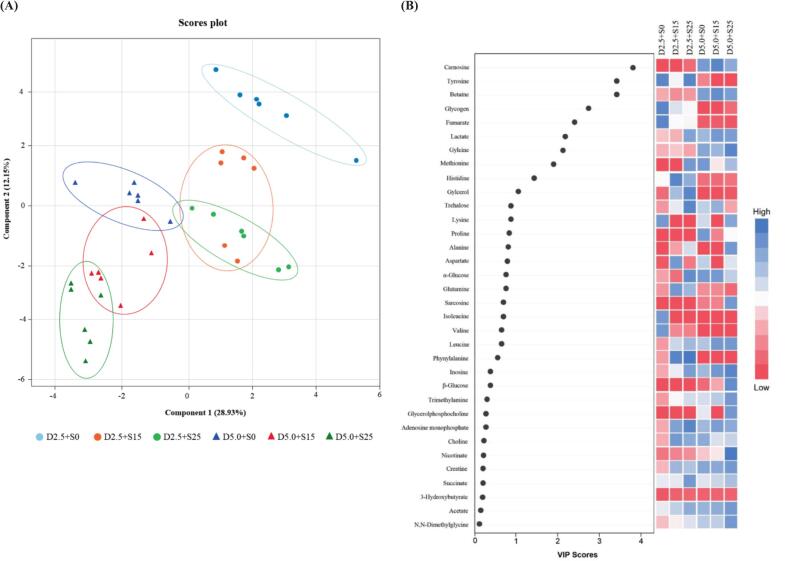
Partial least squares discriminant analysis (PLS-DA) score plot (*R*^*2*^ = 0.9826, *Q*^*2*^ = 0.9805) (**A**) and variable importance in projection (VIP) scores (**B**) of tilapia meat reared under combined dietary salt and water salinity conditions.

Representative spectra of ^1^H NMR metabolite profiles of Nile tilapia fillets under varying water salinity and dietary salt levels are shown in Fig. 2, and the identified metabolites are listed in Table 3**.** A total of 34 metabolites were identified, including 13 amino acids (isoleucine, leucine, valine, alanine, lysine, proline, methionine, glutamine, aspartate, glycine, histidine, tyrosine, phenylalanine), 7 osmolytes (sarcosine, *N*,*N*-dimethylglycine, choline, betaine, glycerolphosphocholine, glycerol, trimethylamine), 6 organic acids (3-hydroxybutyrate, lactate, acetate, succinate, fumarate, nicotinate), 5 energy-related compounds (β-glucose, α-glucose, trehalose, glycogen, creatine), 2 nucleotides (inosine, adenosine monophosphate) and 1 dipeptide (carnosine). Notably, metabolite abundances varied among treatment groups, reflecting physiological responses to different osmotic environments. The results aligned with Hu et al. (2024), who demonstrated metabolic shifts in Nile tilapia under varying culture conditions. In fish reared under the highest salinity condition (D5.0 + S25), lactate, creatine, betaine, glycine, and leucine were the dominant metabolites, with significantly elevated levels compared to the 2.5 % NaCl group (*P* < 0.05). These metabolites are recognized as organic osmolytes that help maintain intracellular osmotic balance without compromising protein integrity (Burg and Ferraris, 2008, Edwards and Marshall, 2012). Their upregulation reflected a robust osmoregulatory response to high salinity, consistent with findings in other euryhaline fish under hyperosmotic stress (Dildar et al., 2025; Huang et al., 2023; Pan et al., 2023). Fish fed with 5.0 % NaCl showed significantly higher levels of both β-glucose and α-glucose compared to those fed lower NaCl levels (*P* < 0.05), indicating elevated blood glucose levels. This, along with increased lactate levels, supported the notion of enhanced glycolytic activity to meet the energy demands of active ion transport under high-salt conditions (Li et al., 2025). Although the dominant metabolites were similar across groups, fish reared at lower salinity (particularly D2.5 + S0) exhibited significantly higher levels of certain metabolites, including the branched-chain amino acids leucine, isoleucine, and valine, as well as succinate (*P* < 0.05). These patterns suggested a metabolic state oriented toward protein turnover and tricarboxylic acid (TCA) cycle activity, reflecting reduced osmotic load and more stable energy metabolism. Additionally, carnosine, a dipeptide with antioxidative and pH-buffering properties, was relatively elevated in these groups. This might contribute to better postmortem muscle preservation, consistent with the observed lower TBARS values and higher meat hardness of fish reared at lower salinity levels. To better understand group differences in metabolites, particularly in relation to metabolic pathways, the identified metabolites were analyzed using PLS-DA, and their contributions to group separation were ranked by VIP scores, as shown in Fig. 3A and Fig. 3B, respectively. The PLS-DA model explained 28.93 % and 12.15 % of the total variance along PC1 and PC2, respectively, with *R*^*2*^ = 0.9826 and *Q*^*2*^ = 0.9805, indicating excellent model reliability and predictability. PC1 primarily separated the samples based on dietary salt levels, with the 2.5 % NaCl groups appearing on the right and the 5.0 % NaCl groups on the left, suggesting that dietary salt had a greater influence on metabolite profiles than water salinity. PC2 captured variation related to water salinity, distinguishing fish reared at 0 ppt (upper area) from those at 15 and 25 ppt (lower area), which showed more similar metabolite patterns. Remarkably, the most distinct separation in the PLS-DA plot was between D2.5 + S0 and D5.0 + S25, which occupied opposite quadrants. This confirmed that these two treatments elicited the most divergent metabolic responses, likely due to the contrast between minimal and maximal osmotic stress. These findings aligned with physiological results, including elevated blood glucose and urea levels in high-salinity groups and reduced muscle protein content, suggesting shifts in energy metabolism and nitrogen mobilization under stress (Gan et al., 2016; Li et al., 2025; Su et al., 2023). Based on VIP scores, carnosine (3.81) contributed most to group separation, followed by tyrosine (3.40), betaine (3.38), and glycogen (2.70). As VIP scores >1 indicated strong influence on group differentiation, six additional metabolites, fumarate, lactate, glycine, methionine, histidine, and glycerol, were also identified, totaling ten key compounds. These metabolites were closely associated with osmoregulation, energy metabolism, and muscle homeostasis, reflecting physiological adaptations to salinity-induced stress (Pan et al., 2023; Su et al., 2023; Zhou et al., 2024). Carnosine acts as an antioxidant and pH buffer, with elevated levels indicating protection against oxidative stress (Li et al., 2025). Tyrosine supports neuroendocrine regulation under osmotic stress, while betaine functions as an osmoprotectant by stabilizing cell volume and protein structure (Huang et al., 2023). Changes in glycogen reflect altered carbohydrate metabolism to meet increased energy demands (Pan et al., 2023; Zhou et al., 2024). Fumarate, a TCA cycle intermediate, signals enhanced mitochondrial activity, while elevated lactate suggests a shift toward anaerobic glycolysis due to energy demand exceeding aerobic capacity (Peng et al., 2025). Glycine contributes to both osmoregulation and antioxidant defense. Together, these metabolite shifts reflect coordinated adaptations to maintain homeostasis and highlight potential biomarkers for muscle quality and stress resilience in tilapia reared under saline conditions. While our discussion focused on the 10 VIP metabolites that most strongly contributed to group separation, the remaining 24 detected metabolites showed relatively low discriminatory power, providing a fuller picture of metabolic adaptation. For instance, glutamine, inosine, and adenosine monophosphate, compounds associated with sweetness and umami taste in meat (Indriani et al., 2024), had relatively low VIP scores, suggesting that salinity-induced metabolic changes did not markedly alter the natural taste of tilapia. This was consistent with the absence of significant differences in sensory flavor-liking scores among treatments (Table 2).

**Table 3:** Identified metabolite contents (ppm) of tilapia meat reared under combined dietary salt and water salinity conditions.

**No.**	**Metabolites**	**Chemical shifts (ppm) and multiplicity**⁎	**D2.5 + S0**	**D2.5 + S15**	**D2.5 + S25**	**D5.0 + S0**	**D5.0 + S15**	**D5.0 + S25**
1	Isoleucine	0.90(t), 1.00(d), 1.29(br. s), 1.48(m), 1.95(m), 3.65(d)	1.02 ± 0.23	ND	ND	ND	ND	ND
2	Leucine	0.96(t), 1.71(m), 3.72(m)	2.38 ± 0.20^d^	5.86 ± 0.26^c^	7.05 ± 0.51^b^	6.54 ± 0.33^b^	6.65 ± 0.32^b^	8.45 ± 0.24^a^
3	Valine	1.03(d), 1.05(d), 2.26(m), 3.59(d)	2.02 ± 0.20^a^	0.45 ± 0.15^b^	0.40 ± 0.08^b^	ND	ND	ND
4	3-Hydroxybutyrate	1.18(d), 2.36(d), 2.44(dd)	0.49 ± 0.09^b^	1.04 ± 0.14^a^	1.26 ± 0.19^a^	1.02 ± 0.15^a^	1.10 ± 0.08^a^	1.05 ± 0.24^a^
5	Lactate	1.32(d), 4.13(q)	16.55 ± 1.01^c^	14.44 ± 0.55^d^	37.80 ± 1.70^a^	33.38 ± 0.62^b^	39.93 ± 1.04^a^	38.93 ± 2.08^a^
6	Alanine	1.47(d), 3.76(q)	ND	0.15 ± 0.08^b^	0.32 ± 0.06^a^	ND	ND	0.48 ± 0.12^a^
7	Lysine	1.40(m), 1.73(m), 1.88(m), 3.74(t)	0.44 ± 0.12	ND	ND	0.30 ± 0.10	ND	0.41 ± 0.06
8	Acetate	1.98(s)	4.60 ± 0.20^d^	5.43 ± 0.34^c^	6.73 ± 0.33^b^	6.42 ± 0.26^b^	6.78 ± 0.40^b^	7.51 ± 0.35^a^
9	Proline	2.03(br. s), 2.05(br. s), 2.34(m), 3.36(t)	ND	ND	ND	1.06 ± 0.07^a^	0.22 ± 0.05^c^	0.58 ± 0.07^b^
10	Methionine	2.16(m), 2.63(t), 3.86(d)	ND	ND	3.18 ± 0.20^a^	3.02 ± 0.22^a^	1.50 ± 0.15^c^	2.44 ± 0.10^b^
11	Succinate	2.41(s)	3.32 ± 0.26^c^	3.40 ± 0.10^c^	5.44 ± 0.25^a^	3.46 ± 0.30^c^	3.79 ± 0.41^bc^	4.37 ± 0.28^b^
12	Glutamine	2.13(m), 2.45(m), 3.78(m)	1.05 ± 0.30^b^	3.88 ± 0.23^a^	3.26 ± 0.29^a^	0.93 ± 0.14^b^	0.88 ± 0.20^b^	0.45 ± 0.08^c^
13	Aspartate	2.06(s), 2.54(dd), 2.71(dd), 4.44(m), 7.96(d)	0.03 ± 0.00^d^	0.69 ± 0.09^a^	0.12 ± 0.04^c^	0.50 ± 0.13^ab^	ND	0.44 ± 0.10^b^
14	Sarcosine	2.74(s), 3.61(s)	ND	ND	ND	0.97 ± 0.11^b^	1.03 ± 0.10^b^	5.05 ± 0.93^a^
15	Trimethylamine	3.23(s)	0.16 ± 0.05^c^	0.32 ± 0.12^b^	0.37 ± 0.08^b^	0.37 ± 0.08^b^	0.42 ± 0.11^ab^	0.56 ± 0.06^a^
16	*N*,*N*-Dimethylglycine	2.93(s), 3.71(s)	3.22 ± 0.24^e^	4.15 ± 0.22^d^	5.26 ± 0.40^c^	6.06 ± 0.29^b^	5.92 ± 0.33^bc^	8.14 ± 0.50^a^
17	Creatine	3.04(s), 3.93(s)	12.08 ± 0.80^e^	26.95 ± 2.00^cd^	25.04 ± 1.05^d^	29.87 ± 0.49^b^	29.27 ± 1.06^bc^	32.12 ± 0.41^a^
18	Choline	3.19(s), 3.51(d), 4.04(m)	0.18 ± 0.04^c^	0.54 ± 0.07^a^	0.49 ± 0.06^ab^	0.49 ± 0.05^ab^	0.35 ± 0.09^b^	0.38 ± 0.06^b^
19	Glycerolphosphocholine	3.21(s), 3.64(m), 3.89(m), 4.29(m)	ND	ND	ND	0.25 ± 0.04^b^	ND	0.40 ± 0.10^a^
20	Betaine	3.27(s), 3.91(s)	7.62 ± 0.20^b^	5.43 ± 0.90^c^	6.26 ± 0.45^c^	21.91 ± 0.44^a^	22.55 ± 0.36^a^	21.88 ± 1.30^a^
21	β-Glucose	4.64(d)	ND	ND	ND	0.44 ± 0.05^c^	1.21 ± 0.21^b^	3.68 ± 0.09^a^
22	α-Glucose	5.25(d)	0.91 ± 0.20^b^	0.40 ± 0.09^c^	2.93 ± 0.35^a^	2.91 ± 0.30^a^	2.89 ± 0.21^a^	2.12 ± 0.74^a^
23	Glycine	3.57(s)	6.12 ± 0.26^d^	7.18 ± 0.22^c^	5.51 ± 0.38^e^	15.89 ± 0.54^b^	15.49 ± 0.35^b^	17.09 ± 0.30^a^
24	Glycerol	3.55(m), 3.63(m), 3.70(m)	0.45 ± 0.06^c^	2.99 ± 0.11^b^	3.68 ± 0.24^a^	0.03 ± 0.00^e^	0.05 ± 0.02^e^	0.16 ± 0.03^d^
25	Histidine	3.21(m), 3.99(t), 7.10(s), 7.90(s)	4.34 ± 0.13^b^	7.93 ± 0.60^a^	7.08 ± 0.35^a^	1.10 ± 0.07^c^	0.97 ± 0.07^c^	1.19 ± 0.10^c^
26	Adenosine monophosphate	4.53(t), 6.11(d), 8.14(s), 8.58(s)	1.85 ± 0.14^d^	5.19 ± 0.25^ab^	5.47 ± 0.29^a^	4.56 ± 0.36^c^	5.33 ± 0.34^ab^	4.95 ± 0.20^ab^
27	Inosine	4.29(m), 4.42(m), 6.11(d), 8.18(s), 8.35(s)	0.61 ± 0.09^e^	1.10 ± 0.10^d^	1.77 ± 0.12^a^	1.51 ± 0.09^b^	1.36 ± 0.08^c^	1.82 ± 0.09^a^
28	Carnosine	2.66(q), 4.46(m), 7.10(s), 8.13(s)	ND	ND	0.62 ± 0.35^b^	4.97 ± 0.50^a^	5.03 ± 0.25^a^	4.55 ± 0.33^a^
29	Trehalose	3.64(m), 3.75(m), 3.83(m), 5.19(s)	0.10 ± 0.02^c^	0.22 ± 0.02^b^	0.36 ± 0.08^a^	0.26 ± 0.06^ab^	0.31 ± 0.05^a^	0.11 ± 0.02^c^
30	Glycogen	3.82(m), 5.33(br. s)	0.40 ± 0.11^a^	0.26 ± 0.09^b^	0.23 ± 0.10^b^	ND	ND	0.05 ± 0.01^c^
31	Fumarate	6.52(s)	2.05 ± 0.20^a^	1.15 ± 0.14^b^	1.09 ± 0.22^b^	0.12 ± 0.03^c^	0.12 ± 0.02^c^	0.08 ± 0.02^c^
32	Tyrosine	1.92(s), 2.87(d), 3.16(d), 4.35(dd), 6.99(d), 7.18(d), 7.74(d)	0.43 ± 0.15^a^	0.25 ± 0.09^b^	0.50 ± 0.09^a^	0.09 ± 0.03^c^	ND	ND
33	Phenylalanine	3.14(m), 3.99(m), 7.39(m)	0.26 ± 0.09^b^	0.88 ± 0.21^a^	1.01 ± 0.20^a^	ND	ND	ND
34	Nicotinate	7.52(m), 8.22(s), 8.83(s), 9.13(s)	0.24 ± 0.05^c^	0.31 ± 0.07^c^	0.36 ± 0.13^c^	0.61 ± 0.10^b^	0.72 ± 0.22^b^	1.45 ± 0.10^a^

Mean ± SD.

ND = Not detected. Different lowercase letters in the same row indicate significant differences (*P* < 0.05).

⁎ Multiplicity: s = singlet; d = doublet; t = triplet; q = quartet; dd = doublet of doublets; m = multiplet; br. s = broad S.

**Fig. 4:**
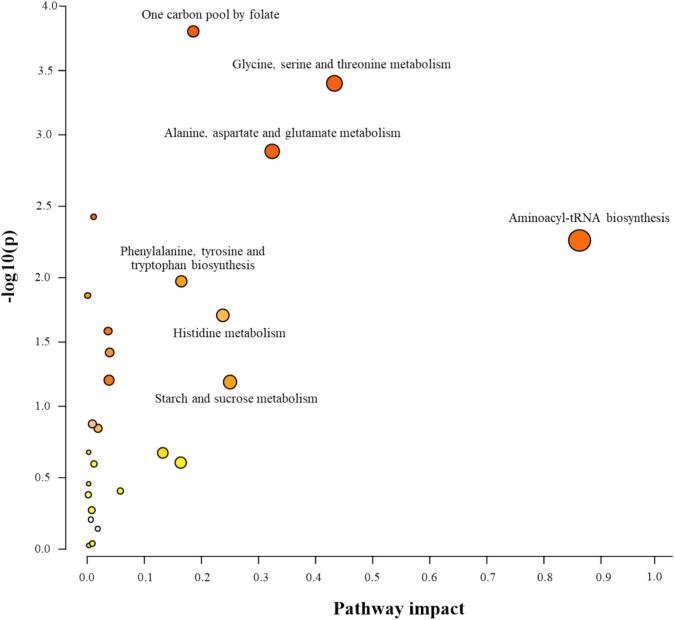
Pathways topology analysis. The color and size of each circle is based on the *P*-value and the pathways impact value, respectively.

Enrichment analysis identified 26 pathways, highlighting significant alterations in key biochemical processes in Nile tilapia under varying salinity and dietary salt conditions (**Table S2**). Among these, seven pathways demonstrated strong statistical relevance, characterized by *P* < 0.05, FDR < 0.05, and impact values >0.1, suggesting meaningful roles in metabolic reprogramming linked to osmotic stress adaptation (Fig. 4, labeled). Aminoacyl-tRNA biosynthesis exhibited the highest impact value (0.8421), indicating major translational shifts driven by elevated protein synthesis demands for cellular repair and stress response under osmotic stress (Petrova et al., 2023). Enrichment of the one carbon pool by folate, as well as glycine, serine, and threonine metabolism highlighted their roles in nucleotide biosynthesis, redox balance, and osmolyte production, crucial for maintaining genomic stability and antioxidant defense under hyperosmotic conditions (Pan et al., 2023; Su et al., 2023). Alterations in alanine, aspartate, and glutamate metabolism (impact = 0.3305) reflected enhanced nitrogen shuttling and energy metabolism, with glutamate and alanine serving dual roles as osmolytes and intermediates in the TCA cycle (Zhang et al., 2024). The enrichment of phenylalanine, tyrosine, and tryptophan biosynthesis, along with histidine metabolism, pointed to increased demand for aromatic amino acids involved in stress signaling and antioxidative functions. Together, these pathways alterations reflected coordinated metabolic reprogramming to support protein turnover, antioxidant defense, and osmotic balance in response to elevated salinity during fish rearing. Notably, starch and sucrose metabolism (impact = 0.2539) was also enriched, suggesting increased mobilization of carbohydrate reserves to meet the elevated energy demands of osmoregulation. These results aligned with observed elevations in glucose and lactate levels, particularly in the D5.0 + S25 group, indicating a shift toward glycolytic energy production under increased salinity. Similar metabolic responses to salinity stress during fish rearing have been reported in previous studies (Pan et al., 2023). These coordinated metabolic shifts enable Nile tilapia to maintain homeostasis under salinity stress, with direct implications for muscle quality and overall aquaculture performance. Particularly, ^1^H NMR-based metabolomics revealed that dietary salt had a stronger influence than water salinity on the muscle metabolic profile, underscoring the importance of feed adaptation with salt prior to rearing under saline conditions. High salinity (25 ppt), combined with 5.0 % NaCl feed adaptation, promoted the accumulation of osmolytes and energy-related metabolites, indicating metabolic stress and adaptive mechanisms to preserve cellular function. The seven enriched metabolic pathways identified (primarily related to amino acid metabolism, carbohydrate metabolism, and energy production) were further consistent with changes in meat quality traits such as pH, lipid oxidation, and texture. This suggested that while the rearing condition may be feasible, it produced meat with distinct characteristics. For example, alterations in amino acid and TCA cycle intermediates (e.g., leucine, isoleucine, fumarate) may affect protein turnover and muscle texture (Pan et al., 2023), whereas shifts in glycolytic and glycogen-related metabolites (e.g., lactate, β-glucose) indicate energy mobilization that can influence postmortem pH and flavor development (Peng et al., 2025). Elevated osmolytes such as betaine and glycine may also enhance oxidative stability and water-holding capacity (Pan et al., 2023), thereby supporting sensory quality. Collectively, these pathway-level changes provide a mechanistic link between salinity-induced metabolic adaptations and the observed alterations in muscle composition and traits (e.g., TVB-N, TBARS, texture) in this study.

### 3.5 PCA

**Fig. 5:**
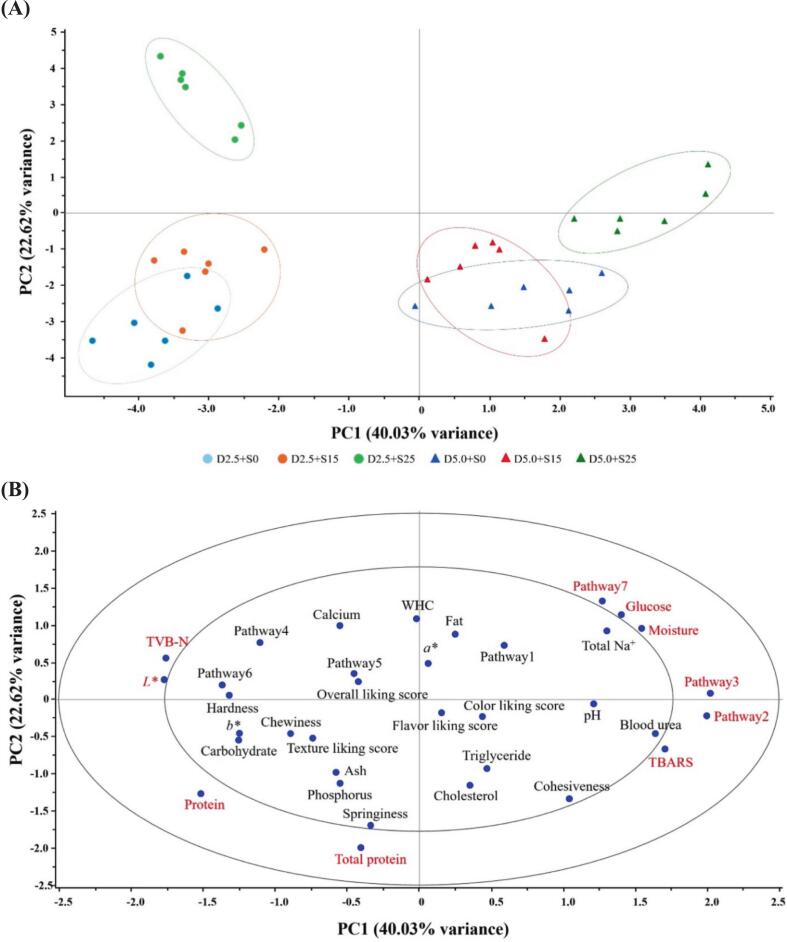
PCA score plot (**A**) and correlation loading plot (PC1 vs. PC2) (**B**)**,** explaining 62.65 % of the total variance, based on differential metabolic pathways, blood biochemistry profile, meat quality traits, and sensory scores of tilapia meat reared under combined dietary salt and water salinity conditions. Red letters indicate a significant level of *P* < 0.05. Pathway1: Aminoacyl-tRNA biosynthesis; Pathway2: One carbon pool by folate; Pathway3: Glycine, serine and threonine metabolism; Pathway4: Alanine, aspartate and glutamate metabolism; Pathway5: Phenylalanine, tyrosine and tryptophan biosynthesis; Pathway6: Histidine metabolism; Pathway7: Starch and sucrose metabolism. (For interpretation of the references to color in this figure legend, the reader is referred to the web version of this article.)

PCA analysis (Fig. 5) provided an integrated multivariate view of how differential metabolic pathways, blood biochemistry, meat quality traits, and sensory attributes varied among Nile tilapia groups exposed to different dietary salt and water salinity. The score plot (Fig. 5A), explaining 62.65 % of the total variance, showed clear group separation. PC1 (40.03 %) distinguished the 5.0 % NaCl groups (right side) from the 2.5 % groups (left side), indicating a strong influence of dietary salt, consistent with PLS-DA results, which revealed a greater impact of dietary salt than water salinity on both metabolite profiles and quality traits. PC2 (22.62) further separated the 25 ppt salinity group (upper region) from the 0 and 15 ppt groups (lower region), reflecting distinct metabolic and quality-related responses under the most extreme salinity condition. The corresponding correlation loading plot (Fig. 5B) clarified the relationship between variables and treatment responses. On the left side, TVB-N and L* (lightness) clustered closely, showing negative correlation with PC1 and an association with lower salinity conditions, particularly D2.5 + S0 sample. This suggested slower post-mortem changes, mainly driven by microbial activity, leading to lower volatile base compound formation and lighter muscle color under minimal osmotic stress, reflecting typical tilapia meat quality with a mild odor and white, lean appearance. Muscle protein content and total blood protein were positively correlated with the 2.5 % NaCl groups, suggesting that under lower osmotic stress, fish retained more muscle protein rather than utilizing it for stress adaptation. The results highlighted the nutritional advantage of fish reared under mild salinity, reinforcing their value as a high-quality protein source for human consumption. No metabolic pathways were significantly correlated with mild dietary salt feeding. In contrast, the right side of the loading plot showed a distinct cluster of Pathways 2, 3, and 7 (representing the one‑carbon pool by folate, glycine, serine, and threonine metabolism, and starch and sucrose metabolism, respectively), alongside TBARS, glucose, and moisture content. These variables were positively correlated with high salinity samples, especially D5.0 + S25, indicating elevated oxidative stress, osmotic imbalance, and carbohydrate mobilization under these conditions. The significant enrichment of these pathways suggested that salinity stress reprograms central metabolism to meet elevated demands for energy production, osmolyte synthesis, and cellular repair (Li et al., 2025; Zhou et al., 2024). Specifically, Pathway 2 (one‑carbon pool by folate) supported methylation reactions and nucleotide synthesis, contributing to cellular turnover and redox homeostasis under salinity stress (Petrova et al., 2023). Pathway 3, glycine and serine metabolism, enhanced osmoregulation and antioxidant defenses (Gan et al., 2016), while Pathway 7, starch and sucrose metabolism, reflected increased glycolytic flux and glycogen breakdown, as corresponded with elevated glucose and lactate levels. These metabolic shifts were supported by key VIP-identified metabolites, glycine, betaine, and lactate, which function as osmoprotectants and energy intermediates. Their accumulation might underlie the observed increases in muscle moisture and TBARS, impacting fillet texture, oxidative stability, and sensory perception. Overall, fish fed with 5.0 % NaCl, particularly D5.0 + S25, were distinguished by alterations in three key metabolic pathways, reflecting the fish's attempt to maintain cellular homeostasis under stress conditions, possibly at the expense of muscle quality, particularly in terms of flavor and oxidative stability.

## 4 Conclusion

This study demonstrated that dietary salt had a greater impact than water salinity on the metabolic profile and meat quality of Nile tilapia. Feeding fish with 5.0 % NaCl, particularly under 25 ppt salinity, significantly altered metabolic pathways related to osmoregulation, amino acid metabolism, energy production, and oxidative stress. These changes were closely linked to variations in muscle quality traits, including pH, lipid oxidation, textural, and nutritional composition, resulting in distinct meat characteristics compared to freshwater-reared fish. The findings emphasize the importance of understanding salinity-induced physiological and biochemical adaptations to optimize feeding strategies and salinity management for sustainable aquaculture. Integrating metabolomics with quality assessment offered a robust framework to identify biomarkers and develop production systems that balance fish welfare, product quality, and environmental sustainability.

## CRediT authorship contribution statement

**Samart Sai-Ut:** Writing – original draft, Visualization, Investigation, Formal analysis. **Sarayut Watchasit:** Writing – review & editing, Visualization, Software, Methodology, Investigation, Formal analysis. **Sylvia Indriani:** Writing – review & editing, Visualization, Investigation, Formal analysis. **Nattanan Srisakultiew:** Writing – review & editing, Visualization, Investigation, Formal analysis. **Surintorn Boonanuntanasarn:** Writing – review & editing, Supervision, Resources, Funding acquisition, Conceptualization. **Chatsirin Nakharuthai:** Writing – review & editing, Supervision, Project administration, Methodology, Funding acquisition, Conceptualization. **Passakorn Kingwascharapong:** Writing – review & editing, Supervision, Investigation. **Jaksuma Pongsetkul:** Writing – review & editing, Validation, Project administration, Methodology, Funding acquisition, Data curation, Conceptualization.

## Ethical statement

Animal slaughter was conducted in accordance with the guidelines of the Department of Livestock Development of Thailand and the National Research Council of Thailand (U1–10177-2565) and was approved by the Animal Ethics Committee of Suranaree University of Technology (approval no. SUT-IACUC-4930030/2023). Ethical approval for human subject participation was obtained from the Human Ethics Committee of Suranaree University of Technology (EC-67-189).

## Declaration of competing interest

The authors declare that they have no known competing financial interests or personal relationships that could have appeared to influence the work reported in this paper.

## Data Availability

No data was used for the research described in the article.

## Appendix A Supplementary data

Table S1 Proximate compositions of experimental diets. Table S2 List of metabolic pathways by enrichment analysis of metabolites. Image 1
