# Supplementary material for: 1H NMR metabolomic responses correlated to meat quality of Nile tilapia (Oreochromis niloticus) reared under combined dietary salt and water salinity conditions
Source: Food Chem X. 2025 Nov 3;32:103235. doi: 10.1016/j.fochx.2025.103235 (PMC12634292; doi:10.1016/j.fochx.2025.103235)
Supplement: Supplementary file 1 — Table S1 Proximate compositions of experimental diets. Table S2 List of metabolic pathways by enrichment analysis of metabolites. [file mmc1.docx]

**Table S1** Proximate compositions of experimental diets.

| **Parameter (%)** | **2.5% NaCl (D2.5)** | **5.0% NaCl (D5.0)** |
| --- | --- | --- |
| Moisture | 9.19 ± 0.84 | 8.68 ± 0.06 |
| Protein | 29.14 ± 0.43 | 28.19 ± 0.05 |
| Fat | 3.20 ± 0.022 | 3.19 ± 0.08 |
| Ash | 11.27 ± 0.24 | 13.26 ± 0.33 |
| Fiber | 5.26 ± 0.63 | 5.37 ± 0.32 |
| Nitrogen free extract^*^ | 41.93 ± 1.63 | 41.32 ± 0.46 |

^*^Nitrogen free extract = dry matter (crude protein + crude lipid + crude fiber + ash)

Mean ± SD.

**Table S2** List of metabolic pathways by enrichment analysis of metabolites.

| **Pathways^*^** | **Total^1^** | **Hits^2^** | ***P*-value** | **-Log(p)** | **FDR^3^** | **Impact^4^** |
| --- | --- | --- | --- | --- | --- | --- |
| **Aminoacyl-tRNA biosynthesis** | 48 | 13 | 0.0032 | 2.49 | 0.0404 | 0.8421 |
| [**One carbon pool by folate**](https://www.metaboanalyst.ca/Secure/pathway/PathResultView.xhtml) | 26 | 6 | 0.0002 | 3.80 | 0.0013 | 0.2148 |
| [**Glycine, serine and threonine metabolism**](https://www.metaboanalyst.ca/Secure/pathway/PathResultView.xhtml) | 34 | 6 | 0.0004 | 3.41 | 0.0024 | 0.4336 |
| [**Alanine, aspartate and glutamate metabolism**](https://www.metaboanalyst.ca/Secure/pathway/PathResultView.xhtml) | 27 | 5 | 0.0012 | 2.92 | 0.0301 | 0.3305 |
| [Valine, leucine and isoleucine biosynthesis](https://www.metaboanalyst.ca/Secure/pathway/PathResultView.xhtml) | 8 | 3 | 0.0035 | 2.45 | 0.0177 | 0.0262 |
| [Arginine biosynthesis](https://www.metaboanalyst.ca/Secure/pathway/PathResultView.xhtml) | 14 | 3 | 0.0129 | 1.89 | 0.0460 | 0.0193 |
| [**Phenylalanine, tyrosine and tryptophan biosynthesis**](https://www.metaboanalyst.ca/Secure/pathway/PathResultView.xhtml) | 4 | 2 | 0.0115 | 1.94 | 0.0478 | 0.1689 |
| [**Histidine metabolism**](https://www.metaboanalyst.ca/Secure/pathway/PathResultView.xhtml) | 17 | 3 | 0.0186 | 1.73 | 0.0499 | 0.2366 |
| β-Alanine metabolism | 18 | 3 | 0.0263 | 1.58 | 0.0731 | 0.0436 |
| [Pyruvate metabolism](https://www.metaboanalyst.ca/Secure/pathway/PathResultView.xhtml) | 23 | 3 | 0.0355 | 1.45 | 0.0887 | 0.0367 |
| [Phenylalanine metabolism](https://www.metaboanalyst.ca/Secure/pathway/PathResultView.xhtml) | 8 | 2 | 0.0479 | 1.32 | 0.1088 | 0.0399 |
| [Glyoxylate and dicarboxylate metabolism](https://www.metaboanalyst.ca/Secure/pathway/PathResultView.xhtml) | 32 | 3 | 0.1514 | 0.82 | 0.2365 | 0.0290 |
| [Nicotinate and nicotinamide metabolism](https://www.metaboanalyst.ca/Secure/pathway/PathResultView.xhtml) | 14 | 2 | 0.1585 | 0.80 | 0.3302 | 0.0209 |
| [Neomycin, kanamycin and gentamicin biosynthesis](https://www.metaboanalyst.ca/Secure/pathway/PathResultView.xhtml) | 2 | 1 | 0.2089 | 0.68 | 0.4018 | 0.0167 |
| [Valine, leucine and isoleucine degradation](https://www.metaboanalyst.ca/Secure/pathway/PathResultView.xhtml) | 40 | 3 | 0.2512 | 0.60 | 0.4486 | 0.0179 |
| [**Starch and sucrose metabolism**](https://www.metaboanalyst.ca/Secure/pathway/PathResultView.xhtml) | 17 | 2 | 0.0490 | 1.29 | 0.0492 | 0.2539 |
| [Pantothenate and CoA biosynthesis](https://www.metaboanalyst.ca/Secure/pathway/PathResultView.xhtml) | 20 | 2 | 0.3311 | 0.48 | 0.5519 | 0.0063 |
| [Citrate cycle (TCA cycle)](https://www.metaboanalyst.ca/Secure/pathway/PathResultView.xhtml) | 20 | 2 | 0.3467 | 0.46 | 0.3467 | 0.0026 |
| [Glycolysis or Gluconeogenesis](https://www.metaboanalyst.ca/Secure/pathway/PathResultView.xhtml) | 26 | 2 | 0.3981 | 0.40 | 0.4855 | 0.0492 |
| [Galactose metabolism](https://www.metaboanalyst.ca/Secure/pathway/PathResultView.xhtml) | 27 | 2 | 0.4677 | 0.33 | 0.6496 | 0.0052 |
| [Nitrogen metabolism](https://www.metaboanalyst.ca/Secure/pathway/PathResultView.xhtml) | 6 | 1 | 0.5248 | 0.28 | 0.6905 | 0.0038 |
| Amino sugar and nucleotide sugar metabolism | 32 | 4 | 0.6310 | 0.20 | 0.7887 | 0.0015 |
| Purine metabolism | 62 | 2 | 0.8318 | 0.08 | 0.9902 | 0.0019 |
| Tyrosine metabolism | 6 | 2 | 0.2344 | 0.63 | 0.2664 | 0.1488 |
| Glycerolipid metabolism | 33 | 2 | 0.2630 | 0.58 | 0.2859 | 0.1709 |
| Arginine and proline metabolism | 38 | 3 | 0.8710 | 0.06 | 0.8375 | 0.0023 |

^*^Pathways with *P*-value < 0.05, FDR < 0.05, and impact > 0.1 are marked in boldface.

^1^ The total number of metabolites in the pathway from the library.

^2^ The number of metabolites related to the pathway in the samples.

^3^ False discovery rate.

^4^ The pathway impact value calculated from pathway topology.
